# 6-(Methylsulfonyl) hexyl isothiocyanate as potential chemopreventive agent: molecular and cellular profile in leukaemia cell lines

**DOI:** 10.18632/oncotarget.22902

**Published:** 2017-12-04

**Authors:** Monia Lenzi, Veronica Cocchi, Marco Malaguti, Maria Cristina Barbalace, Silvia Marchionni, Silvana Hrelia, Patrizia Hrelia

**Affiliations:** ^1^ Department of Pharmacy and Biotechnology, Alma Mater Studiorum-University of Bologna, Bologna, Italy; ^2^ Department for Life Quality Studies, Alma Mater Studiorum-University of Bologna, Rimini, Italy; ^3^ Department of Biomedical and Neuromotor Sciences, Alma Mater Studiorum-University of Bologna, Bologna, Italy

**Keywords:** 6-(methylsulfonyl)hexyl isothiocyanate, apoptosis, cell cycle, Jurkat cells, HL-60 cells

## Abstract

Numerous laboratory and epidemiological studies show that the risk of developing several types of cancer can be reduced with the employment of natural substances that act with multiple mechanisms. In this context, an important role is played by the isothiocyanates. Recently, 6-(methylsulfonyl)hexyl isothiocyanate (6-MITC), present in the root of *Wasabia Japonica*, has stimulated the interest of researchers as a chemopreventive agent. In this particular study we have focused on evaluating 6-MITC’s *in vitro* cytotoxic, cytostatic and cytodifferentiating activities, as well as its pro-apoptotic potential. These effects were investigated by way of flow cytometric analysis of Jurkat and HL-60 cells as well as of healthy lymphocytes extracted from the blood of AVIS donors, in order to verify a potential selectivity of action. The results demonstrate that 6-MITC exerts a stronger cytotoxic effect on tumour cells than on healthy cells. The apoptosis induction exerted by 6-MITC on transformed cells is triggered by an extrinsic pathway, as demonstrated by the statistically significant increase in the percentage of cells with activated caspase-8. It was also observed that 6-MITC is able to limit tumour growth by slowing down and blocking the cell cycle of Jurkat and HL-60 cells respectively, in a dose- and time-related manner, while exerting no activity of any kind on the replication of healthy cells. Finally, by measuring the expression levels of CD-14 and CD-15, 6-MITC showed the ability to induce cytodifferentiation of HL-60 cells into macrophage and granulocytic phenotypes.

## INTRODUCTION

In the late 1970’s Dr. Sporn coined the scientific term “chemoprevention” to represent the possible prevention, halting or reversing of the cancerogenic process through the use of synthetic or natural compounds [1–3]. The development and progression of cancer is associated with a series of events, including dysregulation of tumour suppressor genes, cellular differentiation, excessive proliferation and dysfunction of apoptotic genes [4, 5]. In particular, apoptosis, a physiological process of programmed cell death that plays a key role in homeostasis, is suppressed in many cancer cells. Restoring this ability, therefore, is one of the most important strategies for fighting cancer [6–9]. Phytochemicals derived from edible and medicinal plants have been extensively studied for cancer chemoprevention thanks to their demonstrated ability to modulate the enzymes involved in xenobiotic activation/detoxification, to inhibit cell proliferation and/or to induce apoptosis and the differentiation of neoplastic cells [10–13]. A large number of pure compounds and extracts have also been tested in various experimental models due to their long history of human exposure, high tolerability and low toxicity. In fact, a promising chemopreventive agent should act selectively on cancer cells and cause low toxicity in non-transformed cells [14, 15]. In recent years, growing interest has been focused on isothiocyanates (ITCs), the main pharmacologically active ingredients of cruciferous vegetables that are able to modulate a large number of cancer-related targets including cytochrome P450 enzymes, proteins involved in the antioxidant response, tumorigenesis, apoptosis, the cell cycle and metastasis [16, 17].

More recently, another ITC has stimulated the interest of researchers: 6-(methylsulfonyl)hexyl isothiocyanate (6-MITC), present in high concentrations in *Wasabia Japonica* rhizome. *Wasabia Japonica*, better known as Wasabi, is becoming increasingly important in the scientific field due to the presence, at high concentrations, of numerous ITCs including 2, 4, 6 and 8-MITCs. Among them, 6-MITC is the one present at the highest concentration and is the most interesting bioactive compound. Numerous scientific and epidemiological studies have confirmed, for example, its significant anti-inflammatory [18–20] and antioxidant [21–23] properties, leading to its hypothesised use in chemoprevention. The aim of this study, therefore, was to evaluate the potential of 6-MITC as a chemopreventive agent in leukaemia cell lines. More specifically, its antiproliferative and pro-apoptotic effects were analysed in human lymphoblastic leukaemia cells (Jurkat cells) and in human promyelocytic leukaemia cells (HL-60 cells). In addition to evaluating its cytostatic and cytotoxic effects on transformed cells, its selectivity for non-transformed human peripheral blood lymphocytes (PBL) was also tested using the same endpoints.

There are two main apoptotic pathways: the extrinsic (or death receptor) pathway, and the intrinsic (or mitochondrial) pathway that is regulated by the activation or deactivation of BCL-2 family genes [8, 9, 24]. For this reason, possible molecular mechanisms, such as the intrinsic and extrinsic apoptosis pathways, and apoptotic markers regulating certain tumourigenesis and cell proliferation mechanisms, such as p53, BAX/BCL-2 ratio, cytochrome c, cyclin E2 and cyclin D3 levels, were analysed.

Lastly, most tumour cells exhibit an altered ability to mature into adult non-proliferating cells, thus maintaining a high proliferative state. In contrast, the induction of terminal differentiation generates cells with no or limited replicative capacity, which can then be more easily induced towards apoptosis [25–27]. The study, therefore, concluded with an evaluation of 6-MITC’s ability to stimulate differentiation in HL-60 cells, considered an ideal model for investigating this effect [28].

## RESULTS

### Determination of the 6-MITC concentrations used in subsequent experiments/Guava ViaCount assay

The research began with a preliminary study of the cytotoxic and cytostatic potential of 6-MITC by way of a Guava ViaCount assay to determine the concentrations of ITC to be used in subsequent experiments. To this end, the Jurkat and HL-60 cells were treated for 24h, 48h and 72h with 6-MITC at concentrations of 0 to 64μM while the PBL cells were treated for 24h. The results obtained demonstrated that necrosis of the cancer cells was restrained up to 16μM at 24h and up to 8μM at 48h and 72h. Moreover, the viability of the healthy cells at 24h remained greater than 50% at all concentrations tested (data not shown).

### Effect of 6-MITC on viability, apoptosis and necrosis of Jurkat cells, HL-60 cells and PBL/guava nexin assay

On the basis of the results previously obtained, a Guava Nexin assay was conducted to measure the percentage of live, apoptotic and necrotic cells following the 6-MITC 0-16μM treatment for 24h, 48h and 72h of Jurkat cells (Table 1), the 6-MITC 0-32μM treatment for 24h, 48h and 72h of HL-60 cells (Table 2), and, in parallel, the 6-MITC 0-128μM treatment for 24h of PBL (Table 3).

**Table 1:** Percentage of viable, apoptotic and necrotic Jurkat cells treated with 6-MITC for 24h, 48h and 72h

Jurkat		24h			48h			72h	
	viable cells	apoptotic cells	necrotic cells	viable cells	apoptotic cells	necrotic cells	viable cells	apoptotic cells	necrotic cells
**0 μM**	89.7±0.9	7.6±0.6	2.3±0.3	91.4±1.4	6.1±0.8	2.3±0.6	90.0±0.4	4.6±0.5	4.3±0.1
**2 μM**	79.7±3.3	16.1±2.9	3.9±0.3	85.8±1.6	12.8±0.6	3.3±0.8	85.8±1.3	7.7±0.7	6.2±1.2
**4 μM**	77.3±1.6	16.2±1.1	6.4±0.6	79.8±1.6	15.4±1.6	3.3±0.9	77.6±0.7	13.2±0.9	9.0±1.5
**8 μM**	49.6±2.5	25.4±0.9	24.1±2.5	45±4.3	35±2.3	18.3±3.9	48.8±1.8	31.6±3.5	17.4±2.6
**16 μM**	27.8±1.0	30.3±0.8	42.0±0.8						

Data are presented as mean ± SEM of five independent experiments.

**Table 2:** Percentage of viable, apoptotic and necrotic HL-60 cells treated with 6-MITC for 24h, 48h and 72h

HL-60		24h			48h			72h	
	viable cells	apoptotic cells	necrotic cells	viable cells	apoptotic cells	necrotic cells	viable cells	apoptotic cells	necrotic cells
**0μM**	93.7±02	5.3±0.1	1.1±0.1	94.3±0.5	4.7±0.3	1.0±0.4	94.6±0.2	3.8±0.2	1.3±0.1
**2μM**	93.9±0.1	5.2±0.1	0.9±0.1	93.9±0.1	4.9±0.1	1.3±0.1	95.9±0.1	3.1±0.1	1.0±0.2
**4μM**	91.9±0.4	7.1±0.3	1.0±0.2	91.6±0.6	6.8±0.4	1.6±0.2	92.1±8.9	6.3±0.5	1.4±0.2
**8μM**	83.0±0.4	12.7±0.2	3.3±0.2	66.5±1.5	22.6±1.0	10.5±0.8	58.5±1.7	31.5±2.0	10.0±0.4
**16μM**	46.8±0.9	12.6±0.5	39.2±0.8						
**32μM**	33.3±0.5	15.8±0.4	51.1±0.6						

Data are presented as mean ± SEM of five independent experiments.

**Table 3:** Percentage of viable, apoptotic and necrotic PBL cells treated with 6-MITC for 24h, 48h and 72h

PBL		24h	
	viable cells	apoptotic cells	necrotic cells
**0μM**	77.9±0.2	10.6±0.4	10.9±0.7
**2μM**	76.8±0.9	12.3±0.3	11.2±1.0
**4μM**	68.9±1.0	14.3±0.4	15.9±0.3
**8μM**	67.7±1.2	14.7±0.3	15.7±1.6
**16μM**	67.6±0.3	17.0±0.3	15.4±0.2
**32μM**	68.1±1.3	15.9±0.6	15.3±0.8
**64μM**	51.4±0.4	12.2±0.4	36.4±0.6
**128μM**	40.0 ±0.9	11.8±1.0	47.2±0.7

Data are presented as mean ± SEM of five independent experiments

#### Viability

**Figure 1:**
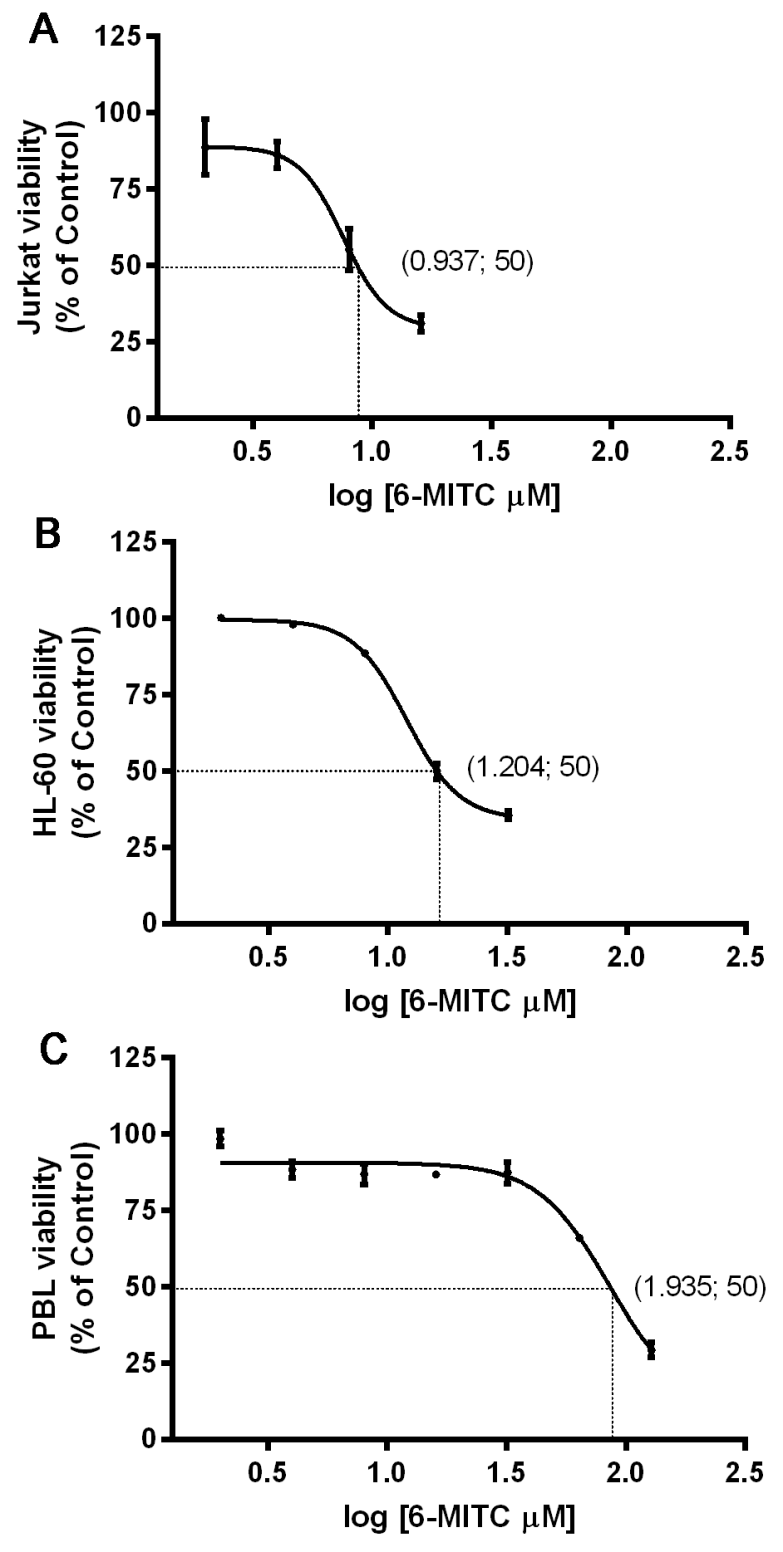
Effect of 6-MITC on viability of Jurkat cells, HL-60 cells and PBL IC_50_ obtained by curve fitting of viable cells after 24h treatment with 6-MITC for Jurkat cells **(A)**, HL-60 cells **(B)** and PBL **(C)**. Data are presented as mean ± SEM of five independent experiments.

The percentage of live cells measured at 24h and normalised to the viability in control cultures (considered to be 100%), was used to obtain the dose-response curve. The IC_50_ value calculated by interpolation was 8.65μM for Jurkat cells, 16μM for HL-60 cells and 86.1μM for PBL, respectively (Figure 1A, 1B, 1C).

The analysis at 48h and 72h allowed confirmation that an acceptable percentage of live Jurkat and HL-60 cells remained at up to 8μM, even after longer treatment times (Table 1 and Table 2).

#### Apoptosis

**Figure 2:**
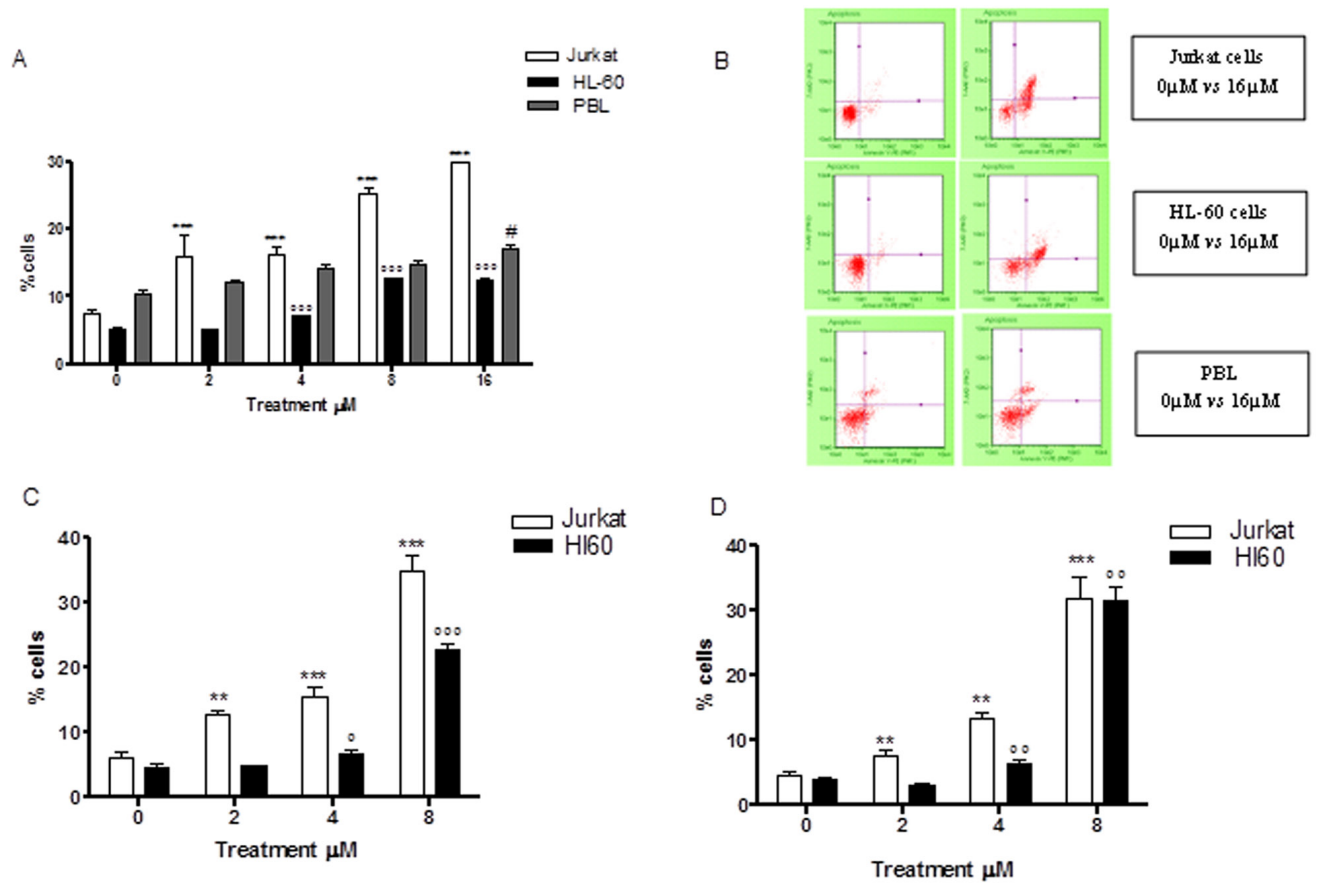
Effect of 6-MITC on apoptosis of Jurkat cells, HL-60 cells and PBL Fraction of apoptotic Jurkat, HL-60 and PBL cells treated with 6-MITC for 24h **(A)** and representative dot plot of apoptosis analysis at 24h treatment **(B)**, fraction of apoptotic Jurkat and HL-60 cells treated with 6-MITC for 48h **(C)** and 72h **(D)**. Apoptosis was evaluated by FCM as described in Methods. Each bar represents the mean ± SEM of five independent experiments. Data were analysed using repeated ANOVA followed by Bonferroni post-test. ^**^p<0.001 *vs* control of Jurkat; ^***^p<0.001 *vs* control of Jurkat; ^°°^ p<0.01 *vs* control of HL-60; ^°°°^ p<0.001 *vs* control of HL-60; ^#^ p<0.05 *vs* control of PBL.

**Figure 3:**
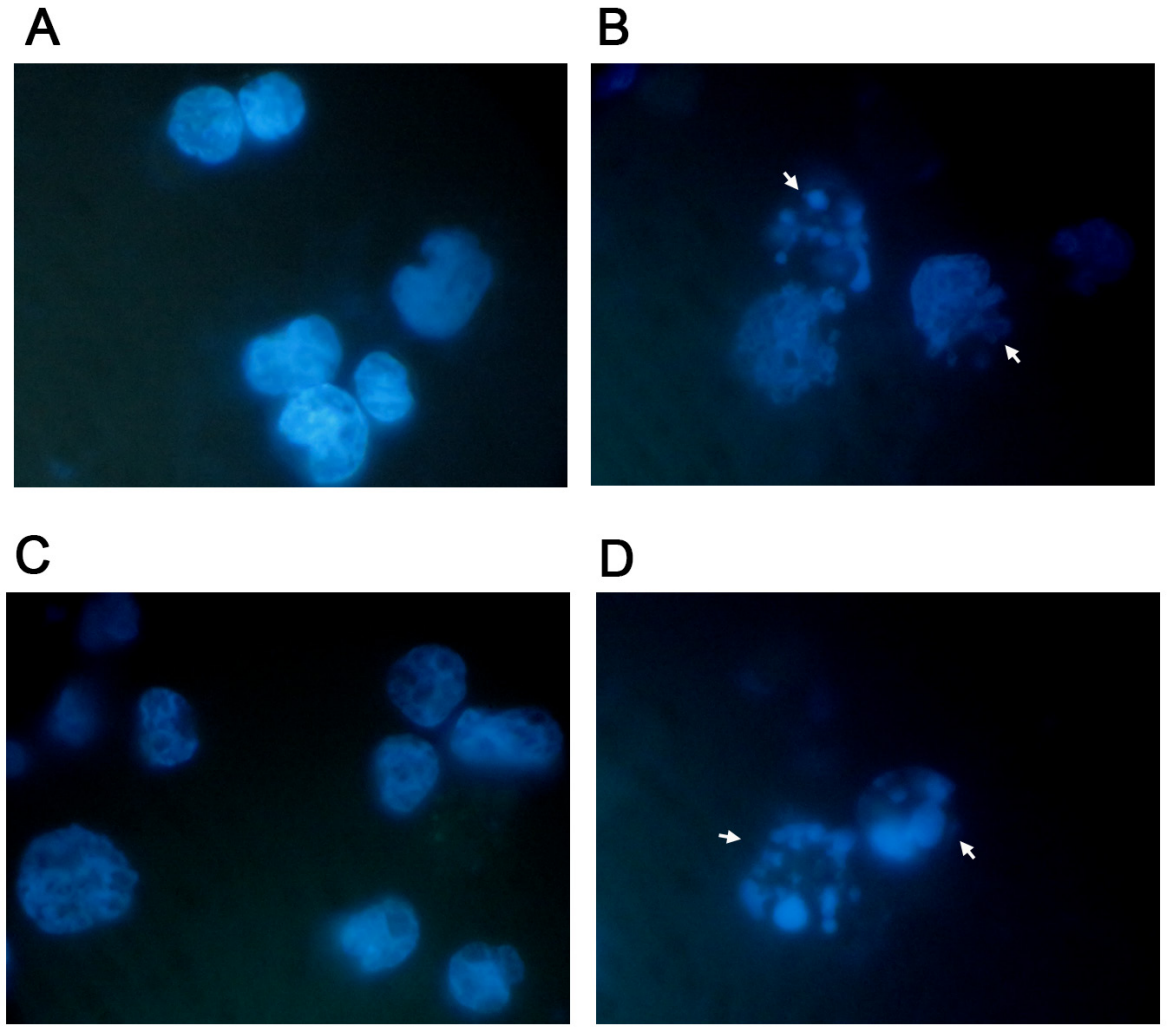
Apoptosis-associated nuclear condensation and fragmentation on Jurkat cells and HL-60 cells Jurkat (A, B) and HL-60 **(B, D)** cells after 72h treatment with 6-MITC 0μM **(A, C)** and 8 μM (B, D) were stained with Hoechst 33258 and evaluated by fluorescence microscopy at 100× magnification as described in Methods. White arrows indicate condensed and/or fragmented nuclei as a marker of apoptosis.

Already at 24h of treatment, Annexin V-PE/7-AAD double staining highlighted a statistically significant increase in Jurkat cells at all concentrations tested. More specifically, with respect to the control cultures at 2μM and 4μM, a population doubling was detected (16.1% *vs* 7.6% and 16.2% *vs* 7.6%), while a 3 and 4 times increase was detected at 8μM and 16μM, respectively (25.4% *vs* 7.6% and 30.3% *vs* 7.6%) (Table 1 and Figure 2A, 2B). A similar pro-apoptotic effect was observed on the HL-60 cells. In fact, the percentage of apoptotic cells increased in a statistically significant manner at a concentration of 4μM (7.1% *vs* 5.2% in controls) and at 8μM (12.7% *vs* 5.2% in controls), while doubling at a concentration of 16μM (12.6% *vs* 5.2% in controls) (Table 2 and Figure 2A, 2B). The induction of apoptosis mediated by 6-MITC on tumour cells was both dose- and time-related. Indeed, a larger increase in the fraction of apoptotic cells was recorded after 48h of treatment than at 24h, while - in Jurkat cells - a 3-times increase was recorded at 4μM (15.4% *vs* 6.1% in controls) and a 6-times increase at 8μM (35.0% *vs* 6.1% in controls) (Table 1 and Figure 2C), and - in HL-60 cells - a 5-times increase was recorded at 8μM (22.6% *vs* 4.8% in controls) (Table 2 and Figure 2C). In addition, after 72h a further 7-times increase of apoptotic cells was recorded in Jurkat cells (31.6% *vs* 4.6% in controls) (Table 1 and Figure 2D) and an 8-times increase recorded in HL-60 cells at the highest concentration tested (31.5% *vs* 3.9% in controls) (Table 2 and Figure 2D). To further confirm the 6-MITC’s pro-apoptotic effect, nuclear condensation and fragmentation were evaluated by fluorescence microscopy (Figure 3).

In order to support the hypothesised selectivity of 6-MITC’s action, we proceeded to similarly analyse its pro-apoptotic potential in PBL. The results showed a statistically significant increase in the percentage of apoptotic cells that only started from a concentration of 16μM (17.0% *vs* 10.6% in controls) and remained constant at 32μM (15.9% *vs* 10.6% in controls). At the highest concentration tested, 64μM, a reduction in apoptotic cells was observed in favour of the necrotic cell fraction, which nonetheless remained below 50% (Table 3 and Figure 2A, 2B). Comparing the results obtained in the different cell lines, it is evident that 6-MITC induces much stronger cytotoxicity on cancer cells than on healthy cells, through stimulation of an apoptotic mechanism (Figure 2A, 2B, 2C, 2D).

#### Necrosis

With regard to necrosis, it is important to underline that the results obtained in Jurkat cells further support the hypothesis of pro-apoptotic activity. In fact, at the 8μM concentration, increasing treatment time resulted in a decrease in the percentage of necrotic cells from 10 to 8 to 4 times, while in HL-60 cells the percentage increased up to 10 times after 48h and then remained steady at 72h (Table 1 and Table 2).

### Evaluation of pro-apoptotic pathway triggered by 6-MITC on Jurkat cells and HL-60 cells/Guava Caspase-8 and Guava MitoPotential assay

In order to assess whether the 6-MITC-induced apoptosis was triggered by the extrinsic or the intrinsic pathway, tumour cells were treated for 24h, 48h, and 72h at concentrations of 4μM and 8μM (<IC_50_ obtained in Jurkat and HL-60 cells).

**Figure 4:**
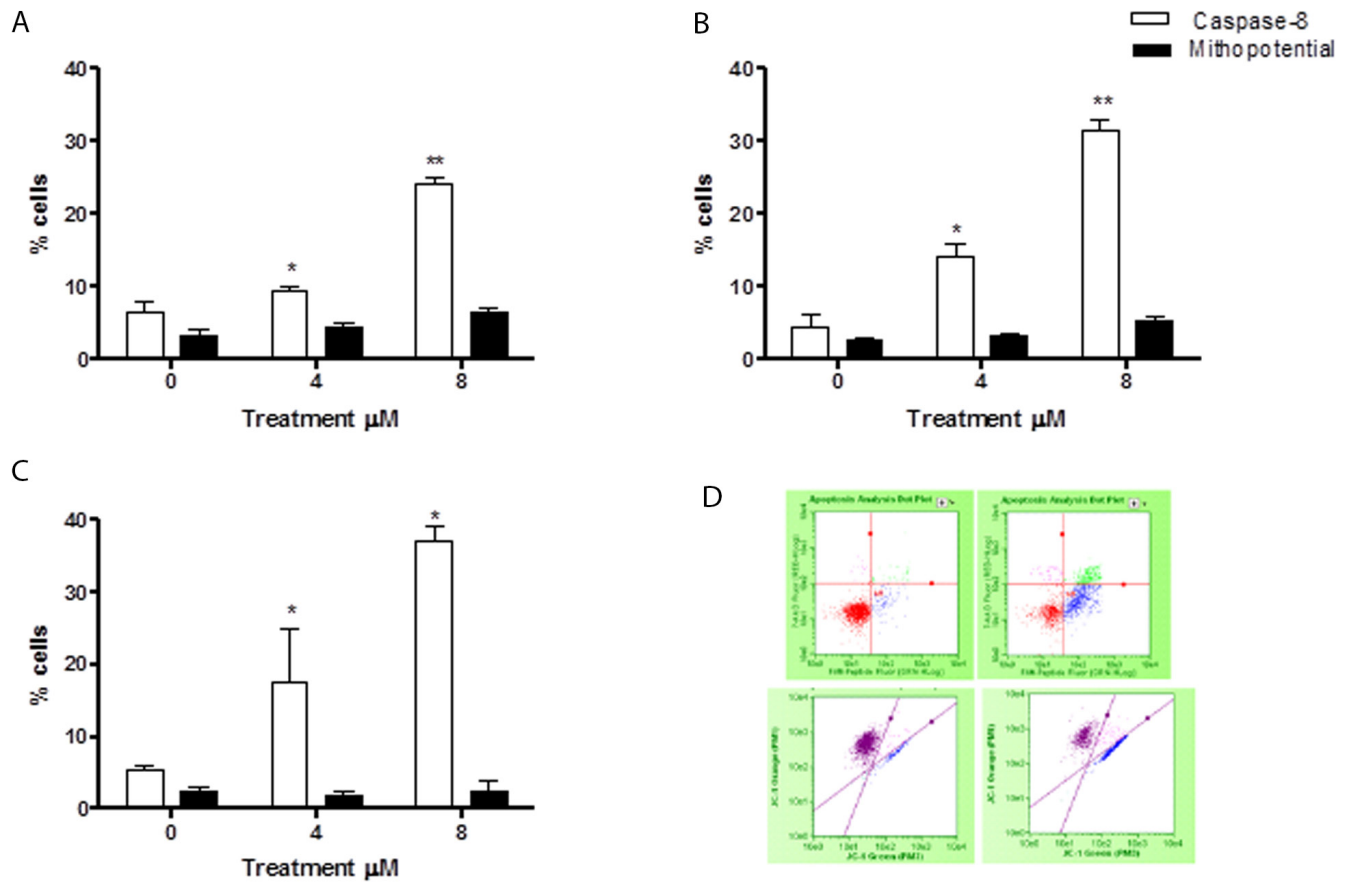
Evaluation of pro-apoptotic pathway triggered by 6-MITC on Jurkat cells Fraction of apoptotic Jurkat cells with active caspase-8 or with altered mitochondrial membrane potential after 24h **(A)**, 48h **(B)**, 72h **(C)** and representative dot plot of caspase-8 (up) and mitochondrial membrane potential (down) analysis at 72h treatment at 0μM (left) and 8μM (right) **(D)**. Active caspase-8 and altered mitochondrial membrane potential was evaluated by FCM as described in Methods. Each bar represents the mean ± SEM of five independent experiments. Data were analysed using repeated ANOVA followed by Bonferroni post-test. ^*^p<0.05 *vs* control; ^**^p<0.01 *vs* control.

**Figure 5:**
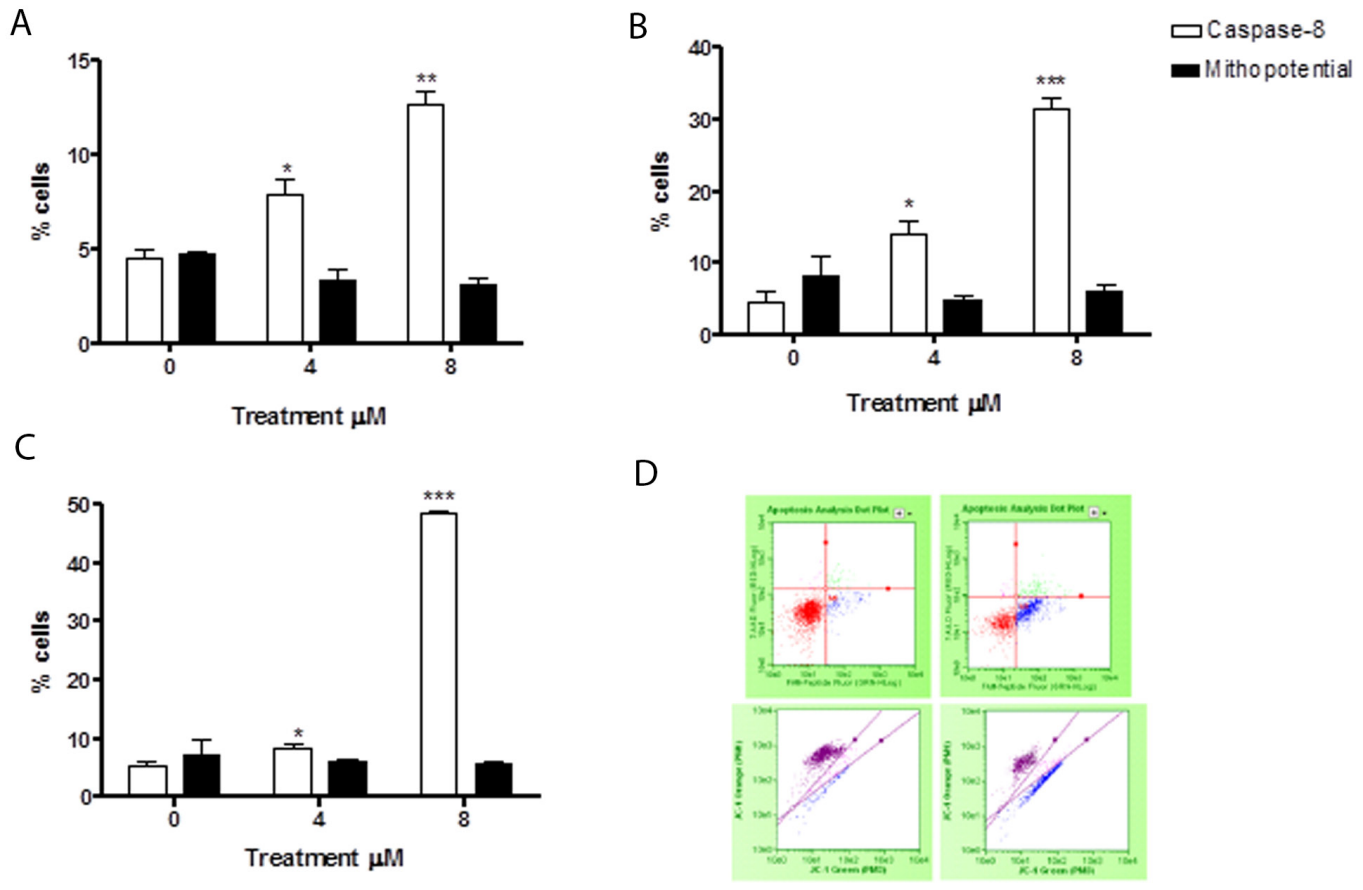
Evaluation of pro-apoptotic pathway triggered by 6-MITC on HL-60 cells Fraction of apoptotic HL-60 cells with active caspase-8 or with altered mitochondrial membrane potential after 24h **(A)**, 48h **(B)**, 72h **(C)** and representative dot plot of caspase-8 (up) and mitochondrial membrane potential (down) analysis at 72h treatment at 0μM (left) and 8μM (right) **(D)**. Active caspase-8 and altered mitochondrial membrane potential was evaluated by FCM as described in Methods. Each bar represents the mean ± SEM of five independent experiments. Data were analysed using repeated ANOVA followed by Bonferroni post-test. ^*^p<0.05 *vs* control; ^**^p<0.01 *vs* control; ^***^ p<0.001 *vs* control.

The levels of cells with activated caspase-8 revealed that the apoptosis induced by 6-MITC was mediated exclusively by extrinsic pathway activation in both cell lines, while the intrinsic pathway did not seem at all involved. In fact, the percentage increase in apoptotic cells with respect to the control cultures measured by the Guava Caspase-8 Assay in Jurkat cells was statistically significant at both concentrations tested at 24h (9.5% and 24.0% *vs* 6.5%), 48h (14.0% and 31.5% *vs* 4.5%) and 72h (17.5% and 37.0% vs 5.5%) (Figure 4A, 4B, 4C, 4D). This percentage increase in the treated versus control cultures was, moreover, fully comparable to that measured in the previously conducted Guava Nexin Assay. For example, after 72h treatment with 8μM the increase was 7 times (Figure 2D and 4C), with a similar result recorded in HL-60 cells. The percentage of activated caspase-8 cells was also statistically higher at both concentrations tested at 24h (7.9% and 12.7% *vs* 4.5% in controls), 48h (7.5% and 27.5% *vs* 5% in controls) and 72h (8.5% and 48.5% *vs* 5.5% in controls) (Figure 5A, 5B, 5C, 5D). Moreover, this percentage was fully comparable to that measured in the previously conducted Guava Nexin Assay, being 8 times higher in the treated versus control cultures after 72h at 8μM (Figure 2D and 5C). In contrast, the fraction of cells with mitochondrial potential depolarisation in treated cultures matched that in control cultures, in both Jurkat and HL-60 cells (Figure 4A, 4B, 4C, 4D; Figure 5A, 5B, 5C, 5D).

### Effect of 6-MITC on cell cycle progression of Jurkat cells, HL-60 cells and PBL/Guava Cell cycle assay

**Figure 6:**
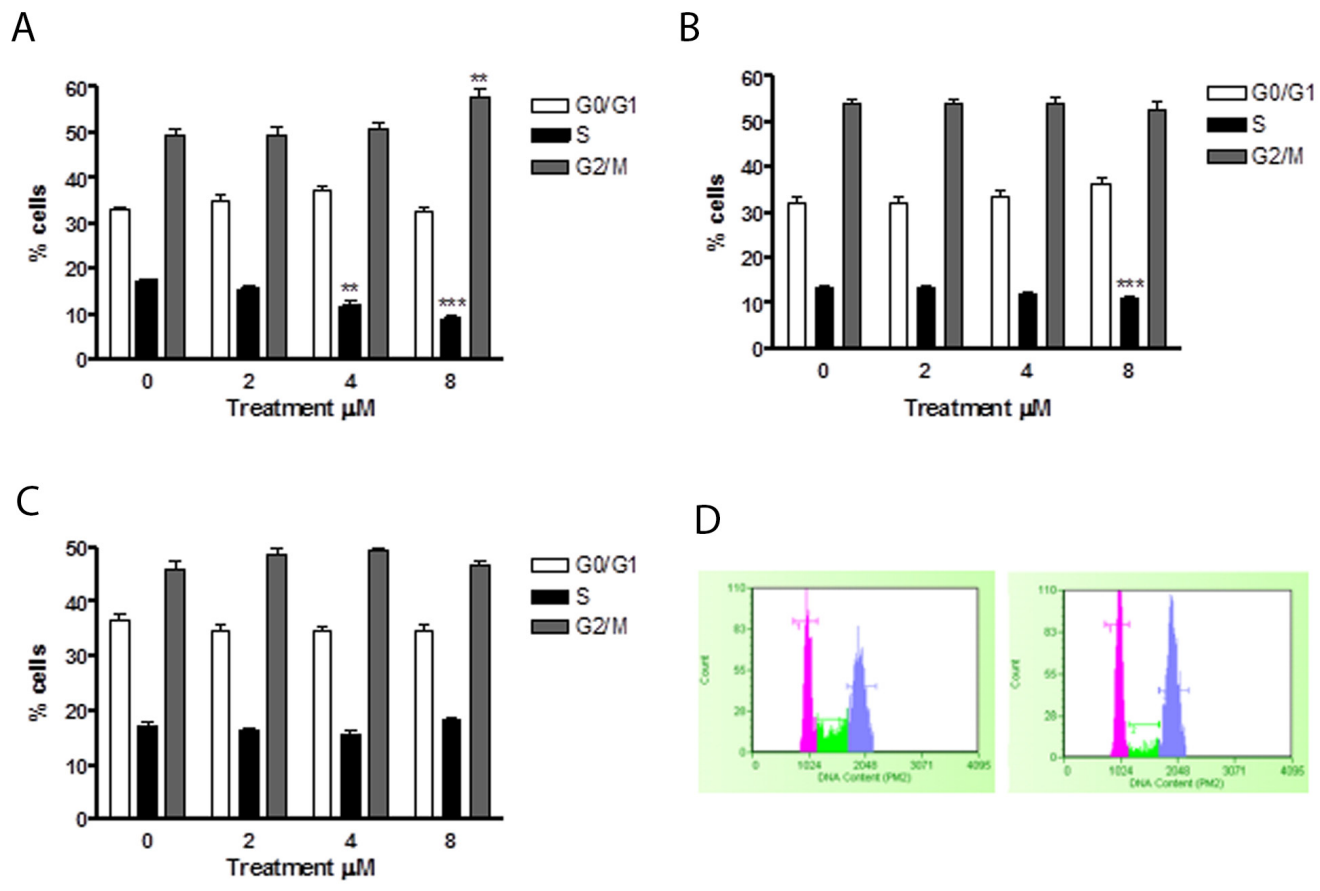
Effect of 6-MITC on cell cycle progression of Jurkat cells Fraction of Jurkat cells in the different phases of the cell cycle after 24h **(A)** and representative histograms of cell cycle analysis at 24h treatment at 0μM (left) and 8μM (right) **(B)** 48h **(C)**, 72h **(D)**. Cellular distribution in the different phases was evaluated by FCM as described in Methods. Each bar represents the mean ± SEM of five independent experiments. Data were analysed using repeated ANOVA followed by Bonferroni post-test. ^**^p<0.01 *vs* control; ^***^p<0.001 *vs* control.

In order to assess whether the apoptosis induced by 6-MITC was an independent event or subsequent to a cell cycle slowdown/block, Jurkat and HL-60 cells were treated with 4μM and 8μM concentrations for 24h, 48h and 72h. PI staining allowed us to highlight the percentage distribution of cells in the different phases of the cell cycle. In particular, after 24h, Jurkat cells demonstrated a statistically significant percentage reduction of cells in the S phase at both 4μM (11.62% *vs* 17.4% in controls) and 8μM (8.8% *vs* 17.4% in controls) (Figure 6A, 6B).

After 48h of treatment, a statistically significant percentage reduction of cells in the S phase was only observed at the higher concentration tested (11.2% *vs* 13.7%) (Figure 6C), while after 72h, no effect on the cell cycle was observed (Figure 6D).

**Figure 7:**
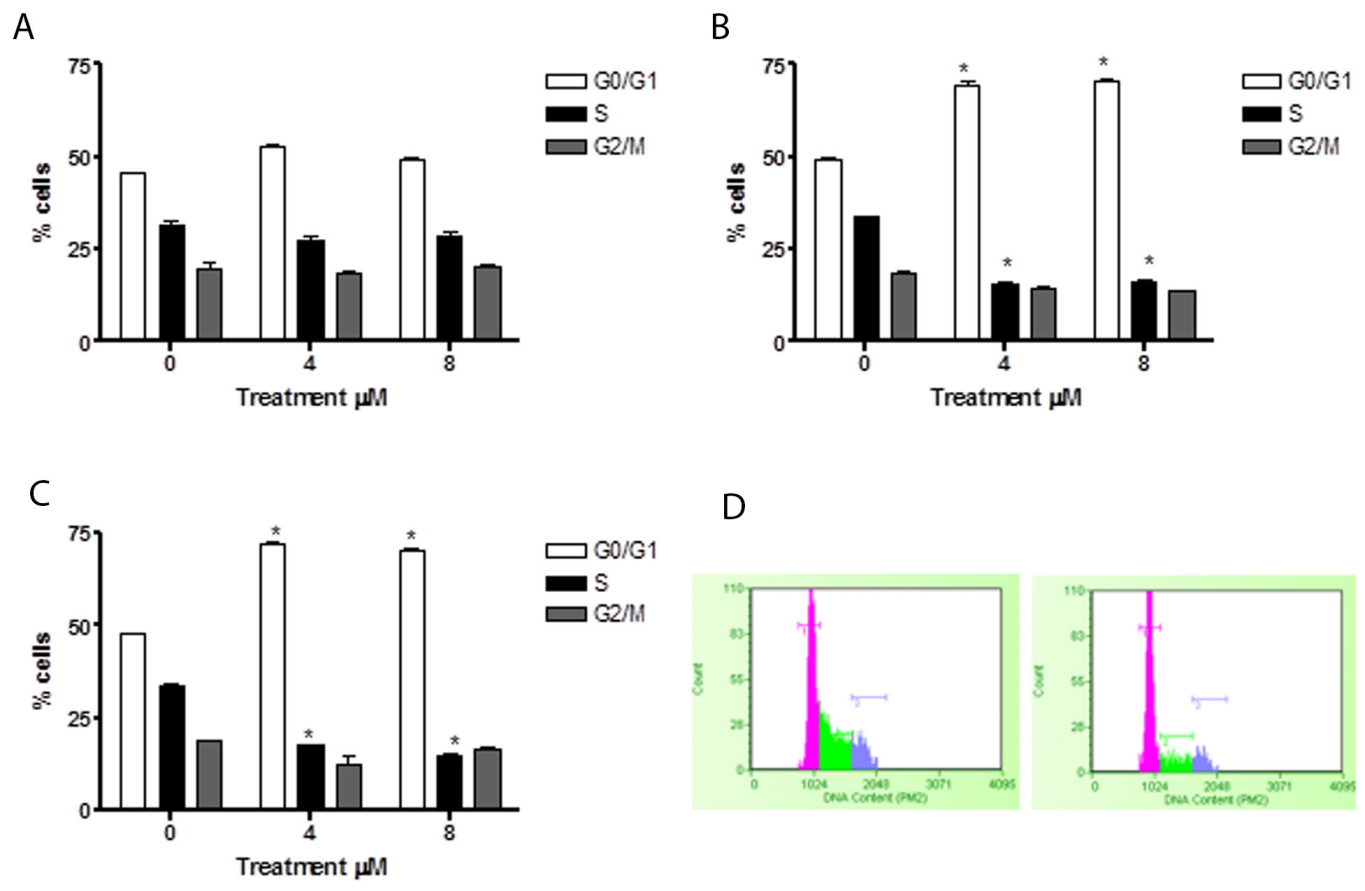
Effect of 6-MITC on cell cycle progression of HL-60 cells Fraction of HL-60 cells in the different phases of the cell cycle after 24h **(A)**, 48h **(B)**, 72h **(C)** and representative histograms of cell cycle analysis at 72h treatment at 0μM (left) and 8μM (right) **(D)**. Cellular distribution in the different phases was evaluated by FCM as described in Methods. Each bar represents the mean ± SEM of five independent experiments. Data were analysed using repeated ANOVA followed by Bonferroni post-test. ^*^p<0.05 *vs* control.

In HL-60 cells, 6-MITC induced no effect after 24h of treatment (Figure 7A), while at 48h and 72h a statistically significant increase of cells in the G_0_/G_1_ phase was observed at 4μM (70.2% and 69.4% *vs* 48.2 %) and 8μM (72.7% and 69.4% *vs* 48.2%) with respect to the control cultures but with no difference in percentage terms between the two treatment times (Figure 7B, 7C, 7D). At the same time, a statistically significant 6-MITC- induced decrease in the S phase was observed at 48h and 72h at concentrations of 4μM (16.4% and 16.5% *vs* 33.4% in controls) and 8μM (17% and 14.6% *vs* 33.4% in controls) (Figure 7B, 7C, 7D).

These results suggest that 6-MITC has the ability to slow down the Jurkat cell cycle and to induce a true block of the HL-60 cell cycle in G_0_/G_1_, in both cases leading to a subsequent decrease in S phase cells.

**Figure 8:**
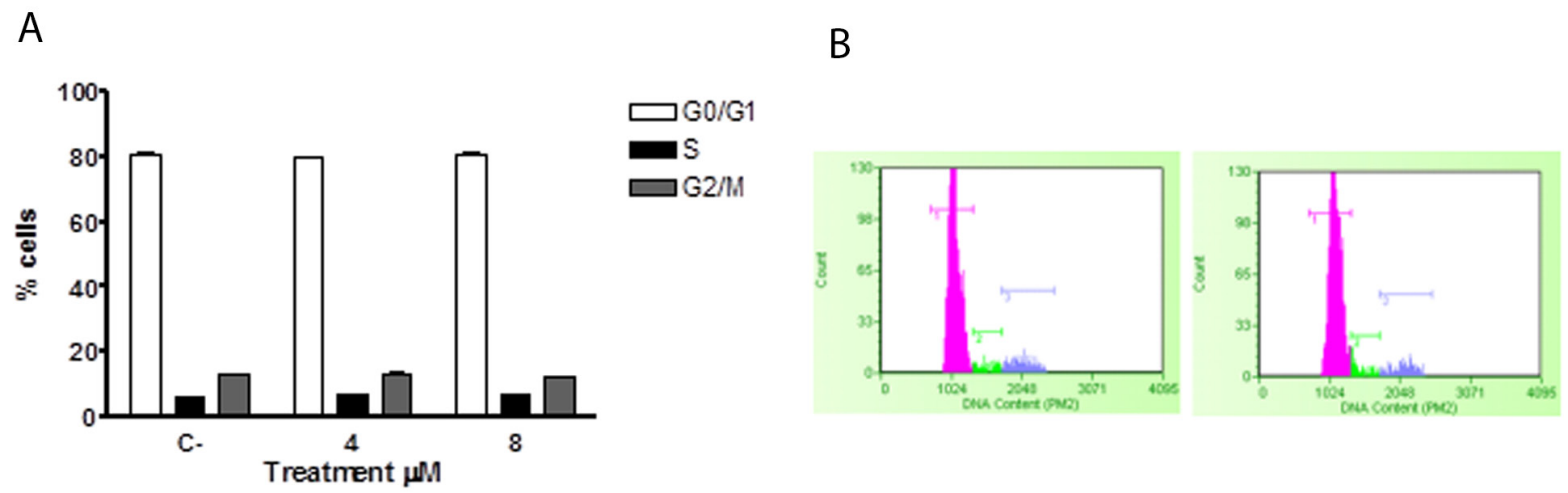
Effect of 6-MITC on cell cycle progression of PBL Fraction of PBL cells in the different phases of the cell cycle after 24 **(A)** and representative histograms of cell cycle analysis at 24h treatment at 0μM (left) and 8μM (right) **(B)**. Cellular distribution in the different phases was evaluated by FCM as described in Methods. Each bar represents the mean ± SEM of five independent experiments. Data were analysed using repeated ANOVA followed by Bonferroni post-test.

Similarly, the potential effects of ITC on the cell cycle of healthy lymphocytes were analysed, with no activity being observed (Figure 8A, 8B).

### Effect of 6-MITC on differentiation of HL-60 cells/analysis of cytodifferentiation

**Figure 9:**
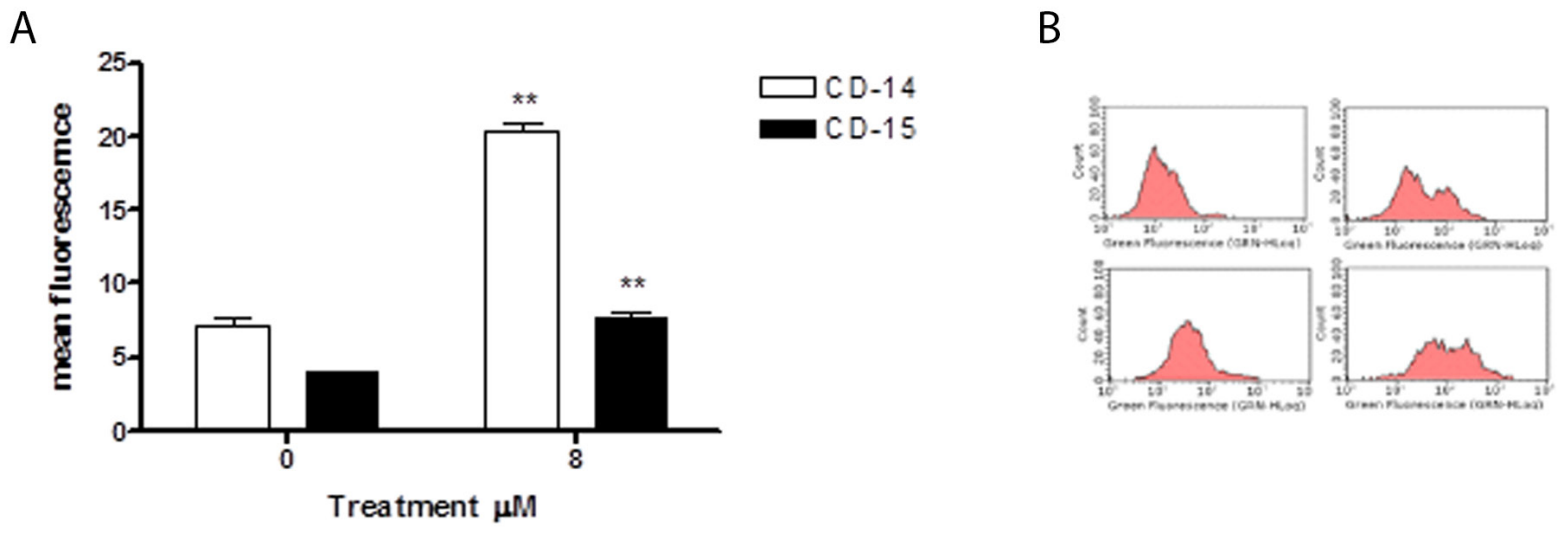
Effect of 6-MITC on differentiation of HL-60 cells CD-14 and CD-15 mean fluorescence intensity after 72h treatment **(A)** and representative dot plot of CD-14 (up) and CD-15 (down) after 72h treatment at 0μM (left) and 8μM (right) **(B)**. CD-14 and CD-15 levels were evaluated by FCM as described in Methods. Each bar represents the mean ± SEM of five independent experiments. Data were analysed using the t-test for paired data. ^**^p<0.001 *vs* control.

We evaluated 6-MITC’s ability to induce cytodifferentiation in HL-60 cells following 24h, 48h and 72h treatment at concentrations of 4μM and 8μM. The results obtained showed that isothiocyanate induces no effect at either concentration after 24h and 48h of treatment (data not shown); however, after 72h of treatment at the higher concentration, it induced a statistically significant increase in differentiated cells of both macrophage and granulocyte phenotypes, more specifically, a 3-times increase in CD-15 positive cells and a 2-times increase in CD-14 positive cells (Figure 9A, 9B).

### Effect of 6-MITC on apoptotic and cell cycle proteins on Jurkat cells and HL-60 cells/analysis of cytochrome C release and cell cycle and apoptotic proteins by flow cytometry (FCM) and by Western immunoblotting (WB)

In order to assess whether the pro-apoptotic and cytostatic ability demonstrated by 6-MITC in Jurkat cells involved modulation of p53 protein, its levels were analysed following treatment at an 8μM concentration for 6h, 24h, 48h and 72h.

**Figure 10:**
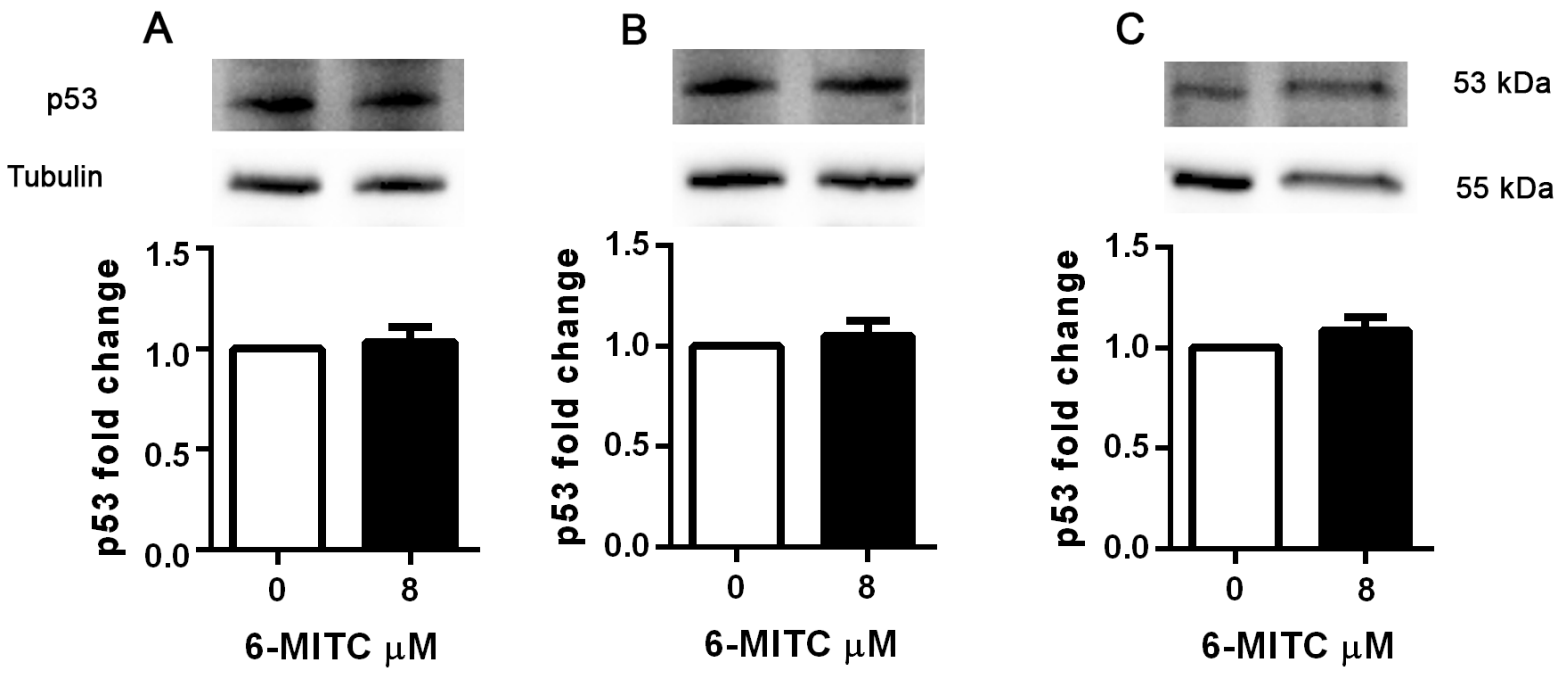
Effect of 6-MITC on p53 protein level in Jurkat cells Effect of 6-MITC’s 8μM treatment on p53 protein expression level in Jurkat cells. Cells were treated for 24h **(A)**, 48h **(B)** and 72h **(C)** with 6-MITC 8μM. Cell lysates were immunoblotted with anti-p53 antibodies as reported in Methods. Results of scanning densitometry analysis performed on three independent autoradiographs are presented. Relative amounts, presented as means ± SEM, were normalized to the intensity of β-tubulin and represented as fold increase *vs* control. Data were analysed using the t-test for paired data. ^*^p<0.05 *vs* control.

As shown in Table 4, the levels of p53 remained unchanged in treated cultures compared to control cultures at all treatment times (Table 4). WB analyses confirmed that 6-MITC’s treatment did not affect p53 protein expression level (Figure 10).

**Table 4:** p53, BAX/BCL-2 ratio, cytochrome c mean fluorescence intensity of Jurkat cells after 24h, 48h and 72h treatment

Jurkat		6h		24h		48h
	0 μM	8μM	0 μM	8μM	0 μM	8μM
**p53**	214.7±1.2	238.2±0.9	341.9±1.7	400.9±0.2	590.7±1.6	608.2±1.4
		**24h**		**48h**		**72h**
	**0**	**8μM**	**0**	**8μM**	**0**	**8μM**
**BAX/BCL-2 ratio**	1.1	0.8	0.9	0.7	0.9	0.4
**Cytochrome**	33.7±0.8	35.8±1.3	32.3±1.3	34.5±0.5	35.9±0.6	29.8±0.8

Protein levels were evaluated by FCM as described in Methods. Data are presented as mean ± SEM of five independent experiments and analysed using the t-test for paired data.

Moreover, in order to check the exclusive involvement of the extrinsic pathway, we also analysed any modulation of the BAX, BCL-2 and cytochrome c proteins in Jurkat and HL-60 cells. The BAX and cytochrome c levels in the treated samples proved perfectly comparable to those of the controls in both cell lines (Table 4 and Table 5). These data corroborate the hypothesis that the pro-apoptotic effect of 6-MITC does not correlate with a loss of mitochondrial transmembrane potential. In contrast, BCL-2 levels increased in the treated cultures with respect to the control cultures, resulting in the BAX/BCL-2 ratio dropping in both cell lines (Table 4 and Table 5).

**Table 5:** BAX/BCL-2 ratio, cytochrome c mean fluorescence intensity of HL-60 cells after 24h, 48h and 72h treatment

HL-60		24h		48h		72h
	0 μM	8μM	0 μM	8μM	0 μM	8μM
**BAX/BCL-2 ratio**	1.1	1.2	0.9	0.5	0.7	0.3
**Cytochrome**	32.8±1.4	34.6±0.5	33.9±0.7	36.1±0.8	34.1±1.1	32.5±1.4

Protein levels were evaluated by FCM as described in Methods. Data are presented as mean ± SEM of five independent experiments and analysed using the t-test for paired data.

**Figure 11:**
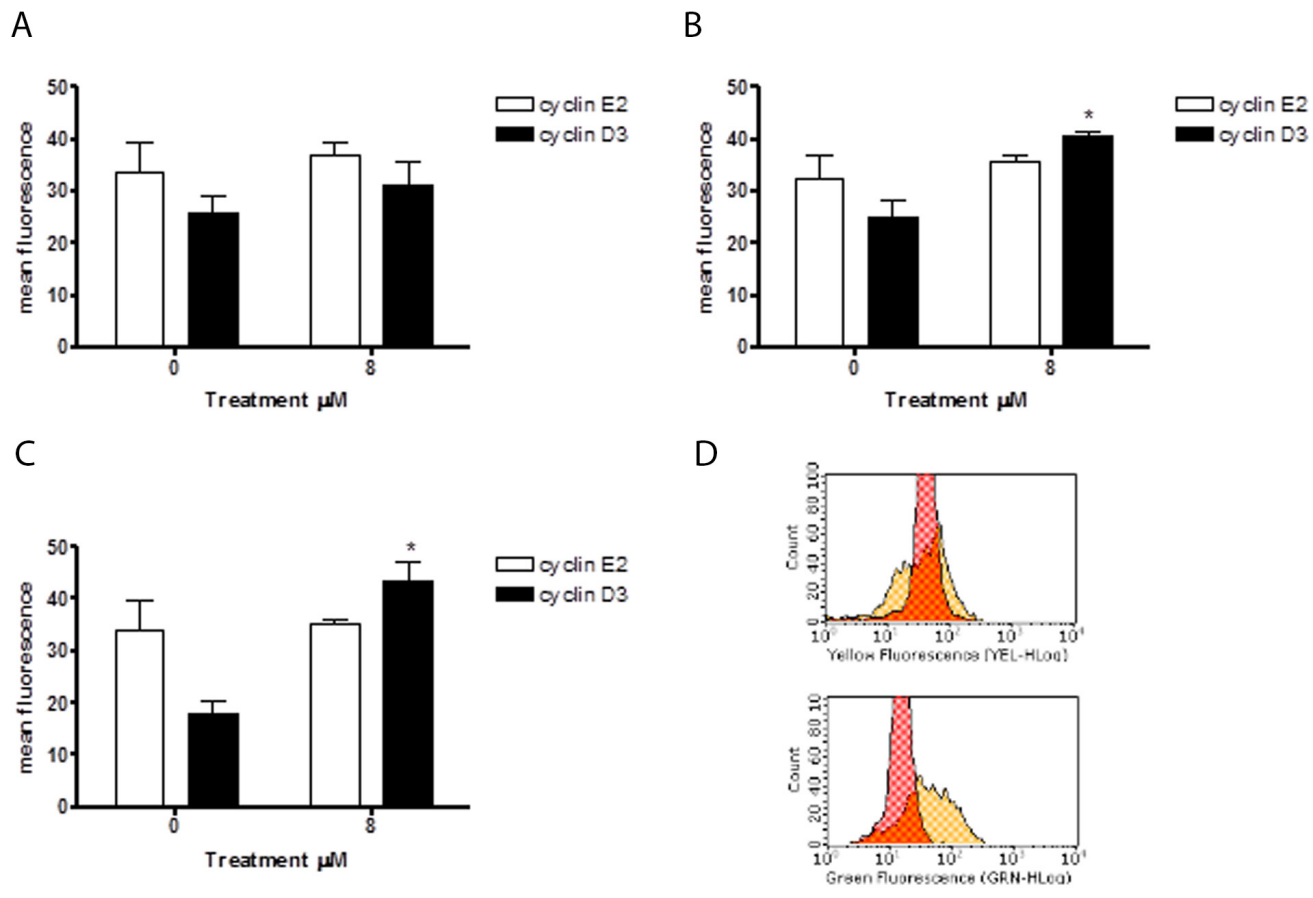
Effect of 6-MITC on cyclin E2 and D3 on HL-60 cells Cyclin E2 and cyclin D3 mean fluorescence intensity after 24h **(A)**, 48h **(B)**, 72h **(C)** 6-MITC’s treatment and representative dot plot of cyclin E2 (up) and cyclin D3 (down) mean fluorescence intensity after 72h treatment **(D)**. Cyclin E2 and cyclin D3 levels were evaluated by FCM as described in Methods. Each bar represents the mean ± SEM of five independent experiments. Data were analysed using the t-test for paired data. ^*^p<0.05 *vs* control.

**Figure 12:**
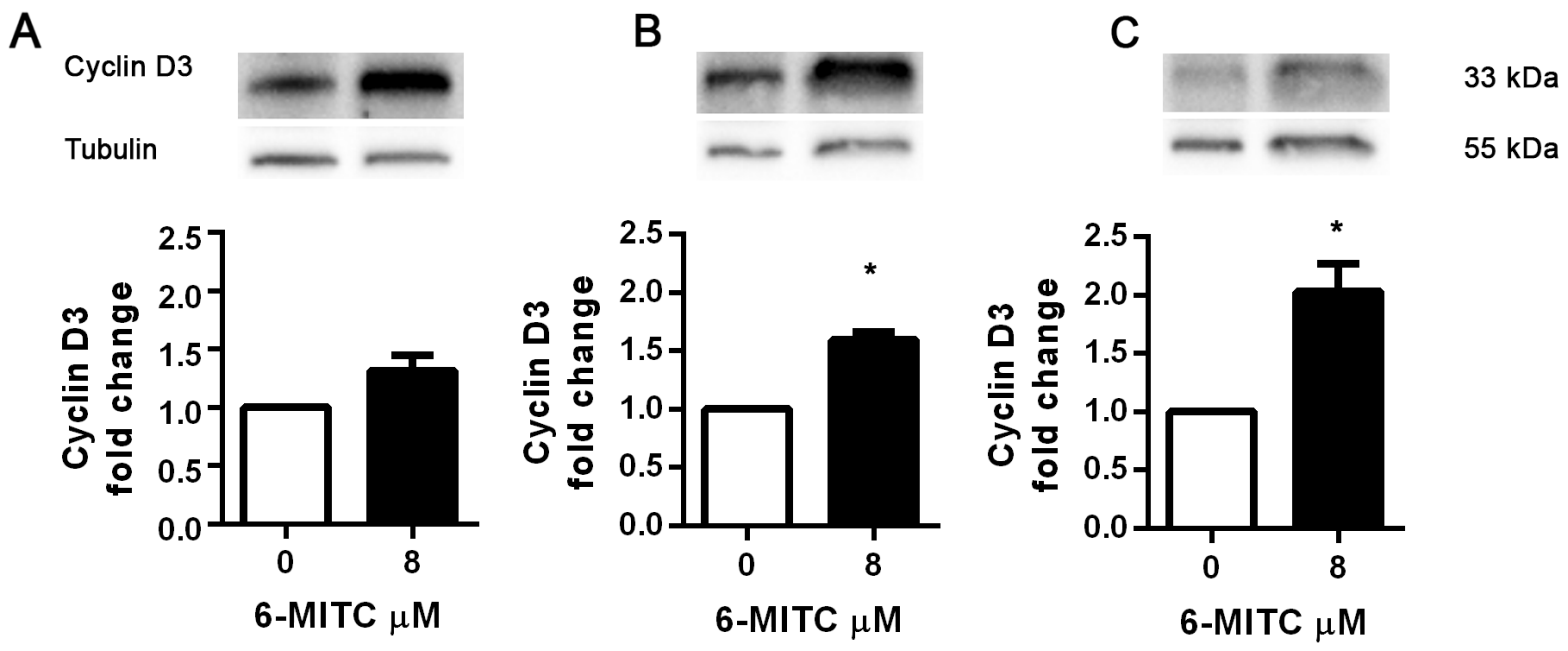
Effect of 6-MITC on cyclin D3 on HL-60 cells Effect of 6-MITC 8μM treatment on cyclin D3 protein expression level in HL-60 cells. Cells were treated for 24h **(A)**, 48h **(B)** and 72h **(C)** with 6-MITC 8μM. Cell lysates were immunoblotted with anti-cyclin D3 antibodies as reported in Methods. Results of scanning densitometry analysis performed on three independent autoradiographs are presented. Relative amounts, presented as means ± SEM, were normalized to the intensity of β-tubulin and reported as fold increase *vs* control. Data were analysed using the t-test for paired data. ^*^p<0.05 *vs* control.

Since 6-MITC was found to arrest the HL-60 cell cycle in the G_0_/G_1_ phase, causing a subsequent decrease of cells in the S phase, we also evaluated its effects in this cell line on the expression of cyclin E2 and the cyclin D3, two proteins involved in the G_1_/S transition phase. As shown in Figure 12, the anti-cyclin E2-PE mean fluorescence intensity remained relatively constant after treatment with 6-MITC at 8μM for 24h, 48h and 72h, while the anti-cyclin D3-FITC mean fluorescence intensity increased at each treatment time, reaching statistical significance at 48h and 72h (Figure 11A, 11B, 11C, 11D). WB analyses of Cyclin D3 protein expression level confirmed this trend (Figure 12).

## DISCUSSION

The aim of this study was to evaluate if 6-MITC elicits chemopreventive activity in leukaemia cell lines.

In recent years there has been an increasing interest in the chemopreventive potential of many naturally occurring substances. In particular, laboratory research and epidemiological studies have shown that the risk of developing various types of cancer may be reduced by the use of compounds that act with multiple mechanisms. Some molecules act early, preventing the activation of pro-carcinogens or favouring the elimination and detoxification of carcinogens through the modulation of biotransformation enzymes; others act on already transformed cells, stimulating apoptosis, arresting/slowing their proliferation or inducing cytodifferentiation, which represent three fundamental mechanisms of chemoprevention [29].

In this context, a major role is exerted by ITCs, a broad group of highly reactive compounds characterised by a common sulphur-containing functional group (-N = C = S) and a variable alkyl or allyl portion. In particular, 6-MITC, the main ITC derived from the rhizome of *Wasabia japonica,* has recently stimulated the interest of researchers through its proven anti-inflammatory, antioxidant and neuroprotective properties that have led to its hypothesised use as a chemopreventive agent [23, 30–32].

In this study, we evaluated the cytotoxic, cytostatic and cytodifferentiating effects of 6-MITC on cancer cells and checked its selectivity of action by monitoring the same effects on healthy cells. The analysis of the specific mechanism of cell death (apoptosis and/or necrosis) demonstrated 6-MITC’s ability to induce apoptosis in a dose- and time- dependent manner in both cell lines tested. In fact, at the highest concentration tested for the longest treatment time, the fraction of apoptotic cells increased 7 times in Jurkat cells and 8 times in HL-60 cells in treated cultures with respect to control cultures. Moreover, statistically significant induction of apoptosis occurred in Jurkat cells at 2μM and in HL-60 cells at 4μM, concentrations that are respectively 8 times and 4 times lower than those required to induce apoptosis in PBL. These results suggest that this ITC possesses an important chemopreventive potential in acting selectively on transformed cells and inducing low toxicity in non-transformed cells; this was confirmed by the observation of an IC_50_ value in PBL more than 10 times higher than that in Jurkat cells and more than 5 times higher than that in HL-60 cells. It is, therefore, possible to define a range of concentrations in which 6-MITC acts selectively.

The apoptotic pathway is generally classified as the intrinsic, mitochondrial, and the extrinsic, death receptor, pathways according to the way of activation. In the extrinsic pathway the activation of death recepror results in the cleavage of pro-caspase-8 and activation of caspase-8 [33]. An analysis of molecular pathways highlighted the interesting capacity of 6-MITC to trigger apoptosis through involvement of the extrinsic pathway, in contrast to many widely studied phytochemicals, such as *Hemidesmus Indicus* [33], or other ITCs, such as SFN [34, 35], phenethyl isothiocyanate [3, 36, 37] and benzyl isothiocyanate [3, 38, 39], which all generally modulate the mitochondrial pathway. In fact, after 72h of treatment at 8μM, the increase in activated caspase-8 apoptotic cells in both cell lines perfectly matched that of the Annexin V-PE positive/7-AAD negative cells previously reported. These data suggest that the pro-apoptotic effect of 6-MITC was due exclusively to the involvement of the extrinsic pathway, since the apoptosis did not seem to correlate with a loss of mitochondrial transmembrane potential. This hypothesis is further corroborated by the comparable number of cells with a depolarised mitochondrial membrane potential observed in the treated and control cultures. BAX and BCL-2 are two proteins located on the mitochondrial membrane. More specifically, BAX exerts pro-apoptotic activity while BCL-2 is an anti-apoptotic protein that inhibits apoptosis and is overexpressed in cancer [40, 41]. Most targeted cancer therapies are based on stimulating the expression of BAX protein and/or suppressing BCL-2 protein. Conversely, in this study the BAX and cytochrome c levels remained unaltered in both the control and treated cultures, while BCL-2 expression was up-regulated in the treated cultures, resulting in a reduced BAX/BCL-2 ratio. This effect could represent a possible attempt at resistance by the cancer cells through a compensatory mechanism in response to the apoptosis induced by the ITC, which is, however, able to induce it by triggering the extrinsic pathway.

6-MITC exhibited antiproliferative effects in both cell lines, as evidenced by the distribution of cells in the different phases of the cell cycle. In particular, it is able to limit Jurkat cell replication by slowing down the cell cycle, causing a resultant reduction in the percentage of S phase cells after 24h of treatment, statistically significant at both tested concentrations; this reduction remained observable after 48h only at the highest concentration tested, and was no longer visible after 72h. These results also suggest a cross-talk between apoptosis and cell cycle regulation. In fact, the different treatment times have allowed us to detect a statistically significant percentage of apoptotic cells at 24h, which continues to increase after 48h and 72h, while the decrease in S phase cells, statistically significant at 24h, gradually disappears as time progresses, so the apoptotic cells observed are either directly induced or those who exit from the cell cycle slowing-down.

6-MITC has also been shown to be able to induce a potent inhibitory effect on HL-60 cell proliferation, resulting in a blockage of the cell cycle’s progression in the G_1_ phase, statistically significant after 48h of treatment and remaining constant after 72h. At the same time, a statistically significant decrease in the S phase was observed. In human cells, cell cycle progression is controlled at three checkpoints - G_1_, S, G_2_/M - by a series of cyclins and cyclin-dependent kinase (CDK) complexes [41–44]. In particular the progression from G0/G1 phase to S phase is regulated by D-type cyclins and E-type cyclins. D-type cyclins acting precociously in the progression through the G1 phase while E-type cyclins become up regulated later during the transition [45]. In the current study, 6-MITC increased the expression of cyclin D3 but did not modulate cyclin E2 levels, A possible explanation could be that 6-MITC blocks HL-60 cell-cycle acting precociously on the G1/S transition and the potential critical factors for G_1_ arrest is cyclin D3.

Recently Wang et al. [46] underpin that the overexpression of cyclins lead sometimes to apoptosis. So we hypothesize that cyclin D3 over-expression might be involved also in 6-MITC-induced apoptosis.

Moreover, the different treatment times also in this case allowed us to hypothesise that the cytotoxic and cytostatic effects exhibited by 6-MITC on HL-60 cells were connected. In fact 6-MITC stimulates apoptosis either as a direct action or indirectly through the blockage of the cell cycle. Further confirming its selectivity of action, is the fact that 6-MITC does not exercise any kind of activity on healthy cell proliferation.

In light of these demonstrated activities, p53 expression levels were analysed in Jurkat cells. p53, in fact, is a tumour suppressor gene that plays an important role in the cell cycle and apoptosis. In non-transformed cells, p53 levels are normally low and rise as a result of DNA damage or other injuries. Its elevation causes a slowdown/blocking of the cell cycle and/or apoptosis induction. However, most tumour cells are p53-mutated or p53-null and, consequently, proliferate indiscriminately and beyond the normal mechanisms of cell survival regulation [9, 47], so making an investigation of this aspect particularly interesting.

Indeed, ITC may be able to modulate the expression of p53 on p53-mutated cells (Jurkat) while, alternatively, exercising its effect without any involvement, as demonstrated in HL-60 cells that are p53-null. Data analysis showed no change in p53 levels at any treatment time, so supporting the hypothesis that ITC induces cytostatic and cytotoxic effects with an independent p53 mechanism.

A large number of malignant cells undergo mitosis, and these cells are poorly differentiated [25, 27]. A chemopreventive agent could act as differentiation inducers, stimulating the differentiation of transformed and immature cells into normal and mature cells. By measuring the expression levels of CD-14 and CD-15 (membrane proteins characteristic of macrophages and granulocytes respectively), 6-MITC demonstrated an ability to induce cytodifferentiation of promyelocytic cells into both macrophage and granulocyte phenotypes after the longer treatment time.

**Figure 13:**
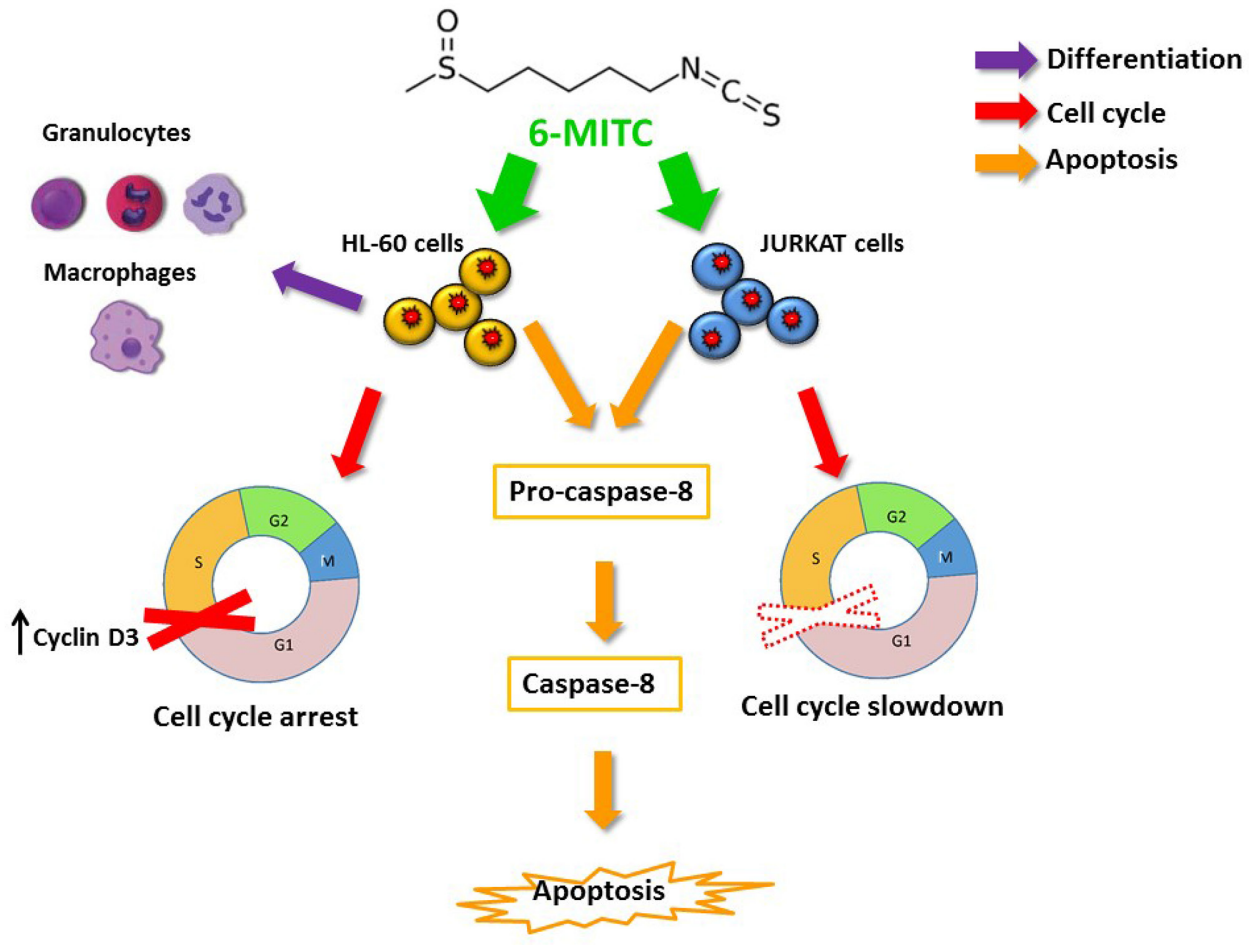
6-MITC inhibits HL-60 and Jurkat cell proliferation via modulating molecular pathways involved in differentiation, cell cycle progression and apoptosis The 6-MITC induced apoptosis on transformed cells results statistically significant at 24 h and dose and time related. Moreover apoptosis is triggered by an extrinsic pathway increasing activated caspase-8 level (orange arrows). 6-MITC is able to limit tumour growth by slowing down the cell cycle of Jurkat cells after 24 h treatment and it blocks HL-60 cell cycle by modulating Cyclin D3 expression level after 48 and 72 h (red arrow). Finally, 6-MITC showed the ability to induce cytodifferentiation of promielocytic HL-60 cells into macrophage and granulocytic phenotypes after 72 h treatment (purple arrow).

Overall, the results obtained and summarised in the table shown in Figure 13 demonstrated that 6-MITC is able to modulate many of the molecular and cellular pathways constituting the main chemopreventive mechanisms.

## MATERIALS AND METHODS

### Reagent

Albumin Bovine (BSA), Anti-β-tubulin, Digitonin, Ethanol, Foetal Bovine Serum (FBS), Formaldehyde, Histopaque-1077, Hoechst 33258 solution, L-Glutamine (L-GLU), Methanol, Penicillin-Streptomycin solution (PS), Phosphate Buffer Saline (PBS), Phytohaemagglutinin (PHA), Roswell Park Memorial Institute (RPMI) 1640 medium, Triton X-100 were purchased from Sigma-Aldrich, St Louis, MO.; Guava Caspase-8 FAM Kit, Guava Cell-cycle Reagent, Guava MitoPotential Kit, Guava Nexin Reagent, Guava ViaCount Reagent were purchased from Merck Millipore, Darmstadt, GER.; Anti-CD-14 (glycosylphosphatidylinositol (GPI)-Linked), anti-CD-15 (3-fucosyl-N-acetyllactosamine (3-FAL)), anti-cyclin D3 were purchased from BioLegend, San Diego, CA.; Anti-cytochrome C, Anti-p53, Anti-BAX were purchased from BD Biosciences, San Jose, CA.; Anti-BCL-2, Anti-cyclin E2 were purchased from Abcam, Cambridge, UK.; Mini–PROTEANTGX Precast Protein Gels, Clarity Western ECL reagent were purchased from BIO-RAD, Hercules, CA, USA; Nitrocellulose membrane was purchased from GE Healthcare, Buckinghamshire, UK.

### 6-MITC

6-MITC was purchased from Abcam, Cambridge, UK. The purity of ITC was >98%. The ITC was dissolved in DMSO up to 97.39 mM stock solution and stored in the dark at -20°C. Increasing concentrations of 6-MITC from 0 to 128μM were tested. DMSO concentration was always in the range 0.05–1% in all the experimental conditions.

### Cell cultures

#### Jurkat

Jurkat cells (acute T lymphoblastic leukaemia) were grown at 37°C and 5% CO_2_ in RPMI-1640 supplemented with 10% FBS, 1% PS, and 1% L-GLU. To maintain exponential growth, the cultures were divided every third day in fresh medium.

The cell density of Jurkat did not exceed the critical value of 3x10^6^ cells/ml of medium and, for every 6-MITC treatment concentration and time, were seeded at 3.75x10^5^ cells/ml.

#### HL-60

HL-60 cells (acute promyelocytic leukaemia) were grown at 37°C and 5% CO_2_ in RPMI-1640 supplemented with 20% FBS, 1% PS, and 1% L-GLU. To maintain exponential growth, the cultures were divided every third day in fresh medium.

To reduce their spontaneous differentiation, the HL-60 cells were never allowed to exceed a density of 1.0x10^6^ cells/ml and, for every 6-MITC’s treatment concentration and time, were seeded at 1.25x10^5^ cells/ml.

#### PBL

Authorization to the use of human blood samples (Buffy coat), for research purposes, has been obtained from AUSL Bologna IT, S. Orsola-Malpighi Hospital - PROT GEN n° 0051937, and informed consent was obtained by AUSL Bologna IT, S. Orsola-Malpighi Hospital from donors for the use of their blood for scientific research purposes.

PBL were isolated using density gradient centrifugation with Histopaque-1077 from the whole peripheral blood of 5 AVIS (Italian Voluntary Blood Donors Association) donors. The donors had the following characteristics: under the age of 35, healthy, non-smoker and with no known exposure to genotoxic chemicals or radiation. The PBL were cultured at 37°C and 5% CO_2_ in RPMI-1640 supplemented with 1% PS, 15% FBS, 1% L-GLU, and 0.5% PHA. The cell density of PBL did not exceed the critical value of 1x10^6^ cells/ml of medium and, for every 6-MITC treatment concentration and time, were seeded at 1.5x10^5^ cells/ml.

### Treatment

Jurkat and HL-60 cells were treated with 0, 2, 4, 8, 16, 32, 64μM of 6-MITC and incubated at 37°C and 5 % CO_2_ for 24, 48, 72h.

After isolation, the PBL were cultured for 44h in complete medium and in the presence of PHA, then treated with 0, 2, 4, 8, 16, 32, 64, 128μM of 6-MITC and incubated at 37°C and 5% CO_2_ for 24h.

### FCM

All FCM analyses were performed using a Guava easyCyte 5HT flow cytometer equipped with a class IIIb laser operating at 488 nm (Merk Millipore, Darmstadt, Germany).

#### Guava ViaCount assay

The cellular density and percentage of viable cells were assessed by FCM and analysed using Guava ViaCount software. After treatment, Guava ViaCount Reagent was added to the cells to discriminate viable from dead cells; the reagent contains the dye propidium iodide (PI), which is only able to penetrate the altered membrane of necrotic cells, bind covalently to the DNA and emit red fluorescence. In contrast, cells with an integral membrane are impermeable to PI and, thus, emit low red fluorescence. The obtained results were expressed as total cells/ml and as the percentage of live cells in treated cultures compared to that in the control cultures.

#### Guava nexin assay

The percentage of apoptotic cells was assessed by FCM and analysed using Guava Nexin software. After treatment, Guava Nexin Reagent was added to the cells: the reagent contains two dyes, 7-aminoactinomycin D (7-AAD) and Annexin-V-PE. As previously described for PI, 7-AAD allows the discrimination between live and dead cells, while Annexin-V-PE allows the identification of apoptotic cells by binding to phosphatidylserine and emitting yellow fluorescence. More specifically, live cells are negative to both 7-AAD and Annexin-V-PE, apoptotic cells are 7-AAD negative and Annexin-V-PE positive, and necrotic cells are positive to both 7-AAD and Annexin-V-PE. The obtained results were expressed as the percentage of apoptotic cells in treated cultures compared to that in the control cultures.

#### Guava caspase-8 assay

The percentage of cells with activated caspase-8 was assessed by FCM and analysed using Guava Caspase software. Guava Caspase-8 Reagent was added to the cells: the reagent contains two dyes, FLICA (an inhibitor of caspase-8) linked to FAM, and 7-AAD. As previously described, 7-AAD allows the discrimination between live and dead cells, while FLICA is cell permeable. Once inside the cell, FLICA binds covalently to the activated caspase-8 and emits green fluorescence. More specifically, live cells are negative to both 7-AAD and FLICA, cells with activated caspase-8 are 7-AAD negative and FLICA positive, and necrotic cells are positive to both 7-AAD and FLICA. The obtained results were expressed as the percentage of cells with activated caspase-8 in treated cultures compared to that in the control cultures.

#### Guava MitoPotential assay

The percentage of apoptotic cells with an altered mitochondrial membrane potential was assessed by FCM and analysed using Guava MitoPotential software. Cells were stained with the Guava MitoPotential Reagent that contains two dyes, JC-1 and 7-AAD. 7-AAD allows the discrimination between live and dead cells, as previously described, while JC-1 is a cell-permeant cationic dye that fluoresces either green or orange depending upon the mitochondrial membrane potential. More specifically, live cells (polarised cells) are 7-AAD negative and orange JC-1 positive, apoptotic cells (depolarised cells) are 7-AAD negative and green JC-1 positive, and necrotic cells are 7-AAD positive and green JC-1 positive. The obtained results were expressed as the percentage of apoptotic cells with an altered mitochondrial membrane potential in treated cultures compared to that in the control cultures.

#### Guava cell cycle assay

The percentage of cells in each stage of the cell cycle was assessed by FCM and analysed using Guava Cell Cycle software. After treatment, cells were fixed and permeabilised with ice-cold 70% ethanol and washed with PBS. The cultures were then suspended in Guava Cell Cycle Reagent that contains the dye PI. PI is able to penetrate the membrane of cells, bind covalently to DNA and emit red fluorescence. More specifically, cells initially in the G_0_/G_1_ phase begin to synthesise DNA in the S phase, until complete duplication in the G_2_/M phase. For this reason, cells in the G_2_/M phase have a double fluorescence compared to those in the G_0_/G_1_ phase, while cells in the S phase have an intermediate fluorescence.

The obtained results were expressed as the percentage of cells in each of the different stages of the cell cycle in treated cultures compared to those in the control cultures.

#### Analysis of cytodifferentiation

The percentage of CD-14 or CD-15 positive cells was assessed by FCM and analysed using Guava InCyte software. Upon conclusion of the treatment time, cells were washed with ice-cold PBS. Cells were then incubated with Anti-CD-14-FITC or Anti-CD-15-FITC and washed. The obtained results were expressed as the percentage of CD-14 and CD-15 positive cells in treated cultures compared to those in the control cultures.

#### Analysis of cytochrome C release

The mean fluorescence intensity value of cytochrome c present was analysed by FCM using Guava InCyte software. Cells were permeabilised with Digitonin (100μg/mL) and fixed in a 4% formaldehyde solution in PBS. Cells were then washed in PBS 1X, incubated in an incubation buffer (0.5g BSA in 100mL PBS 1X) and then incubated overnight at 4°C with anti-cytocrome c monoclonal antibody. At the end of incubation, cells were washed in PBS1X and incubated at room temperature with fluorescein isothiocyanate-labelled secondary antibody. The obtained results were expressed as the mean fluorescence intensity value of cells in treated cultures compared to that in the control cultures. Non-specific binding was excluded by isotype control.

#### Analysis of cell cycle and apoptotic protein

The mean fluorescence intensity value of proteins present was analysed by FCM using Guava InCyte software. Cells were fixed in a 4% formaldehyde solution in PBS and permeabilised with 90% cold methanol. Cells were then incubated with Anti-p53-PE, Anti-BCL-2-FITC, Anti-cyclin E2-PE, Anti-BAX and Anti-cyclin D3 antibodies. The cells (except those stained with BAX and cyclin D3) were washed and analysed. Cells stained with Anti-BAX and Anti-cyclin D3 were washed and incubated with anti-mouse IgG-FITC secondary antibody. The obtained results were expressed as the mean fluorescence intensity value of cells in treated cultures compared to that in the control cultures. Non-specific binding was excluded by isotype control.

### Analysis of apoptosis by fluorescence microscopy

Apoptosis-associated nuclear condensation and fragmentation were evaluated in untreated and treated Jurkat and HL-60 cells by fluorescence microscopy at 100x magnification. 1x10^6^ Jurkat and HL-60 cells were loaded into cytospin chambers and centrifuged ad 450 rpm for 10 minutes. Cells were then fixed in formaldehyde 3.7%, washed in PBS pH 7.2, permeabilised in 0.15% triton X-100 and nuclei were stained with 0.5 μM Hoechst 33258 as reported by Henry et al. [48].

### Analysis of cell cycle and apoptotic protein by WB

Jurkat and HL-60 cell lysates was obtained as previously reported [49], Samples were denatured prior to separation on 4%–20% Mini- PROTEAN TGX Precast Protein Gels. The proteins were transferred to a nitrocellulose membrane at 110 V for 90 min in Tris-glycine buffer. Membranes were then incubated in a blocking buffer containing 5% (w/v) BSA and incubated with anti-p53, anti-Cyclin D3 and anti-β-tubulin, as internal normalizers, overnight at 4°C on an orbital shacker. The results were visualized by chemiluminescence using Clarity Western ECL reagent according to the manufacturer’s protocol (BIO-RAD). Semiquantitative analysis of specific immunolabeled bands was performed using Image Lab 6.0 (BIO-RAD).

### Statistical analysis

All results are expressed as mean ± standard error mean (SEM) of at least five independent experiments. For the statistical analysis of apoptosis, apoptosis pathways and cell cycle we used the Analysis of Variance for paired data (repeated ANOVA), followed by Bonferroni as the post-test. For statistical analyses of cytodifferentiation, protein levels and Western Blotting densitometry we used the t-test for paired data. All the statistical analyses were performed using Prism Software 6.
